# Supplementary material for: Discovery and application of insertion-deletion (INDEL) polymorphisms for QTL mapping of early life-history traits in Atlantic salmon
Source: BMC Genomics. 2010 Mar 8;11:156. doi: 10.1186/1471-2164-11-156 (PMC2838853; doi:10.1186/1471-2164-11-156)
Supplement: Additional file 2 — Information on developed 76 locus single-run INDEL panel in Atlantic salmon. Information on fluorescence labeling, primer concentrations, PCR pooling and links to alignments, INDEL motifs and GENESCAN (Burge and Karlin 1997) predictions of genes/exons are available in html format. [file 1471-2164-11-156-S2.ZIP › Additionalfile2/snpsummary190.html]

```
Cluster 15 Contig 6

prev  Summary    Contig List  next
```

Size of Consensus sequence = 1543

Number of sequences = 114

Minimum redundancy = 6

Key

A gi|117867517|gb|EG940213.1|EG940213 EST\_ssal\_evf\_23760 ssalevf mixed\_tissue Salmo salar cDNA Salmo salar cDNA clone ssal\_evf\_531\_138\_rev 5', mRNA sequence  
B gi|117867518|gb|EG940214.1|EG940214 EST\_ssal\_evf\_23761 ssalevf mixed\_tissue Salmo salar cDNA Salmo salar cDNA clone ssal\_evf\_531\_138\_fwd 3', mRNA sequence  
C gi|24354560|gb|CA048390.1|CA048390 ssalkspa007086 kidney Salmo salar cDNA, mRNA sequence  
D gi|119020410|gb|EG354919.2|EG354919 Ss\_Brain\_42A11\_M13 Brain SSH library Salmo salar cDNA clone Ss\_Brain\_42A11 5', mRNA sequence  
E gi|84203821|gb|DW177517.1|DW177517 SGP304100 Atlantic salmon Brain II cDNA library Salmo salar cDNA clone SS0022D09 5', mRNA sequence  
F gi|29318555|gb|CB506909.1|CB506909 ssaltc022009\_rev reproductive Salmo salar cDNA, mRNA sequence  
G gi|85155144|gb|DW588674.1|DW588674 sbra1-005AE09.b1\_#0033.0 DIAS\_SBRA Salmo salar cDNA 5', mRNA sequence  
H gi|116040793|gb|EG354828.1|EG354828 Ss\_Brain\_43H04\_M13 Brain SSH library Salmo salar cDNA clone Ss\_Brain\_43H04 5', mRNA sequence  
I gi|24339315|gb|CA039009.1|CA039009 ssalnwh007001 whole Salmo salar cDNA, mRNA sequence  
J gi|119020520|gb|EG354828.2|EG354828 Ss\_Brain\_43H04\_M13 Brain SSH library Salmo salar cDNA clone Ss\_Brain\_43H04 5', mRNA sequence  
K gi|89874486|gb|DY730609.1|DY730609 EST\_ssal\_rgb2\_86348 ssalrgb2 mixed\_tissue Salmo salar cDNA Salmo salar cDNA clone ssal\_rgb2\_641\_101\_fwd 3', mRNA sequence  
L gi|24393111|gb|CA062868.1|CA062868 ssalrgb524232 mixed\_tissue Salmo salar cDNA, mRNA sequence  
M gi|26000164|gb|CA770909.1|CA770909 ssalwha001007 whole Salmo salar cDNA, mRNA sequence  
N gi|117470845|gb|EG803064.1|EG803064 EST\_ssal\_evd\_277 ssalevd thymus Salmo salar cDNA Salmo salar cDNA clone ssal\_evd\_002\_055\_rev 5', mRNA sequence  
O gi|89876623|gb|DY732746.1|DY732746 EST\_ssal\_rgb2\_88485 ssalrgb2 mixed\_tissue Salmo salar cDNA Salmo salar cDNA clone ssal\_rgb2\_644\_259\_fwd 3', mRNA sequence  
P gi|117452820|gb|EG785039.1|EG785039 EST\_ssal\_evd\_41319 ssalevd thymus Salmo salar cDNA Salmo salar cDNA clone ssal\_evd\_555\_135\_rev 5', mRNA sequence  
Q gi|85054788|gb|DW582966.1|DW582966 EST\_ssal\_rgb2\_47385 rgb2 Salmo salar cDNA clone ssal\_rgb2\_576\_202\_fwd 3', mRNA sequence  
R gi|117868084|gb|EG940780.1|EG940780 EST\_ssal\_evf\_24271 ssalevf mixed\_tissue Salmo salar cDNA Salmo salar cDNA clone ssal\_evf\_532\_043\_rev 5', mRNA sequence  
S gi|29311516|gb|CB500290.1|CB500290 ssalbrh003047 head Salmo salar cDNA, mRNA sequence  
T gi|117565873|gb|EG886849.1|EG886849 EST\_ssal\_evf\_45065 ssalevf mixed\_tissue Salmo salar cDNA Salmo salar cDNA clone ssal\_evf\_560\_089\_rev 5', mRNA sequence  
U gi|89843908|gb|DY700225.1|DY700225 EST\_ssal\_rgb2\_55964 ssalrgb2 mixed\_tissue Salmo salar cDNA Salmo salar cDNA clone ssal\_rgb2\_590\_171\_fwd 3', mRNA sequence  
V gi|117857608|gb|EG930304.1|EG930304 EST\_ssal\_evf\_32431 ssalevf mixed\_tissue Salmo salar cDNA Salmo salar cDNA clone ssal\_evf\_543\_038\_rev 5', mRNA sequence  
W gi|24344601|gb|CA043681.1|CA043681 ssalplnb510073 gut Salmo salar cDNA, mRNA sequence  
X gi|25998741|gb|CA769486.1|CA769486 ssalrgb508249 mixed\_tissue Salmo salar cDNA, mRNA sequence  
Y gi|29311544|gb|CB500318.1|CB500318 ssalbrh011035\_read0 head Salmo salar cDNA, mRNA sequence  
Z gi|24352116|gb|CA045946.1|CA045946 ssalbrh011035 head Salmo salar cDNA, mRNA sequence  
a gi|117545650|gb|EG877095.1|EG877095 EST\_ssal\_eve\_20746 ssaleve thyroid Salmo salar cDNA Salmo salar cDNA clone ssal\_eve\_528\_079\_rev 5', mRNA sequence  
b gi|117445611|gb|EG777834.1|EG777834 EST\_ssal\_evd\_4434 ssalevd thymus Salmo salar cDNA Salmo salar cDNA clone ssal\_evd\_504\_221\_rev 5', mRNA sequence  
c gi|117531834|gb|EG863279.1|EG863279 EST\_ssal\_eve\_56242 ssaleve thyroid Salmo salar cDNA Salmo salar cDNA clone ssal\_eve\_576\_251\_rev 5', mRNA sequence  
d gi|117541692|gb|EG873137.1|EG873137 EST\_ssal\_eve\_34016 ssaleve thyroid Salmo salar cDNA Salmo salar cDNA clone ssal\_eve\_546\_075\_rev 5', mRNA sequence  
e gi|117545652|gb|EG877097.1|EG877097 EST\_ssal\_eve\_20748 ssaleve thyroid Salmo salar cDNA Salmo salar cDNA clone ssal\_eve\_528\_080\_rev 5', mRNA sequence  
f gi|117856557|gb|EG929253.1|EG929253 EST\_ssal\_evf\_31485 ssalevf mixed\_tissue Salmo salar cDNA Salmo salar cDNA clone ssal\_evf\_541\_310\_rev 5', mRNA sequence  
g gi|117467751|gb|EG799970.1|EG799970 EST\_ssal\_evd\_56756 ssalevd thymus Salmo salar cDNA Salmo salar cDNA clone ssal\_evd\_576\_209\_rev 5', mRNA sequence  
h gi|117425131|gb|EG757355.1|EG757355 EST\_ssal\_sjb\_6023 ssalsjb mixed\_tissue Salmo salar cDNA Salmo salar cDNA clone ssal\_sjb\_013\_311\_rev 5', mRNA sequence  
i gi|45320083|gb|CK890350.1|CK890350 SGP150580 Atlantic salmon Ovaries cDNA library Salmo salar cDNA clone KG5-0555 5', mRNA sequence  
j gi|89857064|gb|DY713187.1|DY713187 EST\_ssal\_rgb2\_68926 ssalrgb2 mixed\_tissue Salmo salar cDNA Salmo salar cDNA clone ssal\_rgb2\_611\_330\_fwd 3', mRNA sequence  
k gi|24387400|gb|CA057157.1|CA057157 ssalrga504114 mixed\_tissue Salmo salar cDNA, mRNA sequence  
l gi|117544417|gb|EG875862.1|EG875862 EST\_ssal\_eve\_19635 ssaleve thyroid Salmo salar cDNA Salmo salar cDNA clone ssal\_eve\_526\_275\_rev 5', mRNA sequence  
m gi|117497068|gb|EG829285.1|EG829285 EST\_ssal\_eve\_41649 ssaleve thyroid Salmo salar cDNA Salmo salar cDNA clone ssal\_eve\_556\_250\_rev 5', mRNA sequence  
n gi|117847758|gb|EG920454.1|EG920454 EST\_ssal\_evf\_59306 ssalevf mixed\_tissue Salmo salar cDNA Salmo salar cDNA clone ssal\_evf\_579\_266\_rev 5', mRNA sequence  
o gi|117523735|gb|EG855462.1|EG855462 EST\_ssal\_eve\_16109 ssaleve thyroid Salmo salar cDNA Salmo salar cDNA clone ssal\_eve\_521\_332\_rev 5', mRNA sequence  
p gi|117856544|gb|EG929240.1|EG929240 EST\_ssal\_evf\_31474 ssalevf mixed\_tissue Salmo salar cDNA Salmo salar cDNA clone ssal\_evf\_541\_304\_rev 5', mRNA sequence  
q gi|24388343|gb|CA058100.1|CA058100 ssalrga513148 mixed\_tissue Salmo salar cDNA, mRNA sequence  
r gi|59833375|gb|DN139058.1|DN139058 SGP263883 Atlantic salmon Head kidney cDNA library Salmo salar cDNA clone FN4-2077 5', mRNA sequence  
s gi|116040839|gb|EG354874.1|EG354874 Ss\_Brain\_31B12\_M13 Brain SSH library Salmo salar cDNA clone Ss\_Brain\_31B12 5', mRNA sequence  
t gi|117452821|gb|EG785040.1|EG785040 EST\_ssal\_evd\_41320 ssalevd thymus Salmo salar cDNA Salmo salar cDNA clone ssal\_evd\_555\_135\_fwd 3', mRNA sequence  
u gi|89880061|gb|DY736184.1|DY736184 EST\_ssal\_rgb2\_91923 ssalrgb2 mixed\_tissue Salmo salar cDNA Salmo salar cDNA clone ssal\_rgb2\_650\_161\_rev 5', mRNA sequence  
v gi|45310513|gb|CK880882.1|CK880882 SGP141203 Atlantic salmon Head kidney cDNA library Salmo salar cDNA clone FN4-0207 5', mRNA sequence  
w gi|50137173|gb|CO470866.1|CO470866 SGP212585 Atlantic salmon Eye cDNA library Salmo salar cDNA clone OY4-2429 5', mRNA sequence  
x gi|45303789|gb|CK874158.1|CK874158 SGP135353 Atlantic salmon Brain cDNA library Salmo salar cDNA clone HJ5-0895 5', mRNA sequence  
y gi|89857063|gb|DY713186.1|DY713186 EST\_ssal\_rgb2\_68925 ssalrgb2 mixed\_tissue Salmo salar cDNA Salmo salar cDNA clone ssal\_rgb2\_611\_330\_rev 5', mRNA sequence  
z gi|84205396|gb|DW178360.1|DW178360 SGP305090 Atlantic salmon Brain II cDNA library Salmo salar cDNA clone SS0154E09 5', mRNA sequence  
A gi|85155148|gb|DW588675.1|DW588675 sbra1-005AE09.g1\_#0021.0 DIAS\_SBRA Salmo salar cDNA 5', mRNA sequence  
B gi|116040853|gb|EG354888.1|EG354888 Ss\_Brain\_31E03\_M13 Brain SSH library Salmo salar cDNA clone Ss\_Brain\_31E03 5', mRNA sequence  
C gi|119020999|gb|EG354874.2|EG354874 Ss\_Brain\_31B12\_M13 Brain SSH library Salmo salar cDNA clone Ss\_Brain\_31B12 5', mRNA sequence  
D gi|117857609|gb|EG930305.1|EG930305 EST\_ssal\_evf\_32432 ssalevf mixed\_tissue Salmo salar cDNA Salmo salar cDNA clone ssal\_evf\_543\_038\_fwd 3', mRNA sequence  
E gi|84206839|gb|DW179221.1|DW179221 SGP306139 Atlantic salmon Brain II cDNA library Salmo salar cDNA clone SS0190G04 5', mRNA sequence  
F gi|84206483|gb|DW179010.1|DW179010 SGP305871 Atlantic salmon Brain II cDNA library Salmo salar cDNA clone SS0182G04 5', mRNA sequence  
G gi|70786143|gb|DR695783.1|DR695783 SMT3-0105 Atlantic Salmon macrophage - Aeromonas in vitro infection Salmo salar cDNA clone SMT3-0105, mRNA sequence  
H gi|84204840|gb|DW178046.1|DW178046 SGP304710 Atlantic salmon Brain II cDNA library Salmo salar cDNA clone SS0135C04 5', mRNA sequence  
I gi|45320755|gb|CK891022.1|CK891022 SGP163283 Atlantic salmon Ovaries cDNA library Salmo salar cDNA clone KG4-0611 5', mRNA sequence  
J gi|40541752|gb|BM414014.1|BM414014 EST00936 Atlantic salmon Lambda Zap Express ovary cDNA library Salmo salar cDNA clone oyr\_002\_g09 5' similar to Stathmin, CAA46450, mRNA sequence  
K gi|40542300|gb|BU693987.1|BU693987 EST0024 Atlantic salmon Lambda Zap Express eye cDNA library Salmo salar cDNA clone EEA2-a07 5' similar to Stathmin, mRNA sequence  
L gi|89874487|gb|DY730610.1|DY730610 EST\_ssal\_rgb2\_86349 ssalrgb2 mixed\_tissue Salmo salar cDNA Salmo salar cDNA clone ssal\_rgb2\_641\_101\_rev 5', mRNA sequence  
M gi|89883359|gb|DY739482.1|DY739482 EST\_ssal\_rgb2\_95221 ssalrgb2 mixed\_tissue Salmo salar cDNA Salmo salar cDNA clone ssal\_rgb2\_655\_375\_rev 5', mRNA sequence  
N gi|84205144|gb|DW178219.1|DW178219 SGP304910 Atlantic salmon Brain II cDNA library Salmo salar cDNA clone SS0148E11 5', mRNA sequence  
O gi|84204951|gb|DW178106.1|DW178106 SGP304777 Atlantic salmon Brain II cDNA library Salmo salar cDNA clone SS0139F09 5', mRNA sequence  
P gi|84208432|gb|DW180039.1|DW180039 SGP303651 Atlantic salmon Gills II cDNA library Salmo salar cDNA clone SS0106F08 5', mRNA sequence  
Q gi|84205166|gb|DW178232.1|DW178232 SGP304925 Atlantic salmon Brain II cDNA library Salmo salar cDNA clone SS0149E01 5', mRNA sequence  
R gi|84206263|gb|DW178900.1|DW178900 SGP305729 Atlantic salmon Brain II cDNA library Salmo salar cDNA clone SS0179F05 5', mRNA sequence  
S gi|84209317|gb|DW180486.1|DW180486 SGP306386 Atlantic salmon Heart II cDNA library Salmo salar cDNA clone SS0012H04 5', mRNA sequence  
T gi|84210019|gb|DW180809.1|DW180809 SGP306737 Atlantic salmon Heart II cDNA library Salmo salar cDNA clone SS0031G08 5', mRNA sequence  
U gi|84205894|gb|DW178651.1|DW178651 SGP305420 Atlantic salmon Brain II cDNA library Salmo salar cDNA clone SS0170F09 5', mRNA sequence  
V gi|84206869|gb|DW179236.1|DW179236 SGP306158 Atlantic salmon Brain II cDNA library Salmo salar cDNA clone SS0191C08 5', mRNA sequence  
W gi|85155524|gb|DW588806.1|DW588806 sbra1-006AC06.y1\_#0549.2 DIAS\_SBRA Salmo salar cDNA 5', mRNA sequence  
X gi|45328765|gb|CK899032.1|CK899032 SGP162620 Atlantic salmon Testis cDNA library Salmo salar cDNA clone MG4-1351 5', mRNA sequence  
Y gi|83639033|gb|DW005623.1|DW005623 SGP280095 Atlantic salmon Brain cDNA library Salmo salar cDNA clone HJ4-1991 5', mRNA sequence  
Z gi|45304707|gb|CK875076.1|CK875076 SGP136298 Atlantic salmon Brain cDNA library Salmo salar cDNA clone HJ4-1328 5', mRNA sequence  
a gi|29324576|gb|CB513350.1|CB513350 ssalrgb508249\_rev mixed\_tissue Salmo salar cDNA, mRNA sequence  
b gi|45327248|gb|CK897515.1|CK897515 SGP154681 Atlantic salmon Testis cDNA library Salmo salar cDNA clone MG5-0272 5', mRNA sequence  
c gi|45303606|gb|CK873975.1|CK873975 SGP135154 Atlantic salmon Brain cDNA library Salmo salar cDNA clone HJ5-0668 5', mRNA sequence  
d gi|117298520|gb|EG648333.1|EG648333 SGP317919 Atlantic salmon normalized Brain cDNA library Salmo salar cDNA clone BRAIN3-A8 5', mRNA sequence  
e gi|119021186|gb|EG354888.2|EG354888 Ss\_Brain\_31E03\_M13 Brain SSH library Salmo salar cDNA clone Ss\_Brain\_31E03 5', mRNA sequence  
f gi|57124773|gb|CX356214.1|CX356214 ssalrgb514163\_rev\_0 mixed\_tissue Salmo salar cDNA, mRNA sequence  
g gi|89876622|gb|DY732745.1|DY732745 EST\_ssal\_rgb2\_88484 ssalrgb2 mixed\_tissue Salmo salar cDNA Salmo salar cDNA clone ssal\_rgb2\_644\_259\_rev 5', mRNA sequence  
h gi|89843906|gb|DY700224.1|DY700224 EST\_ssal\_rgb2\_55963 ssalrgb2 mixed\_tissue Salmo salar cDNA Salmo salar cDNA clone ssal\_rgb2\_590\_171\_rev 5', mRNA sequence  
i gi|85054787|gb|DW582965.1|DW582965 EST\_ssal\_rgb2\_47384 rgb2 Salmo salar cDNA clone ssal\_rgb2\_576\_202\_rev 5', mRNA sequence  
j gi|45310757|gb|CK881126.1|CK881126 SGP141453 Atlantic salmon Head kidney cDNA library Salmo salar cDNA clone FN4-0502 5', mRNA sequence  
k gi|84204156|gb|DW177686.1|DW177686 SGP304290 Atlantic salmon Brain II cDNA library Salmo salar cDNA clone SS0084B03 5', mRNA sequence  
l gi|117846961|gb|EG919657.1|EG919657 EST\_ssal\_evf\_58588 ssalevf mixed\_tissue Salmo salar cDNA Salmo salar cDNA clone ssal\_evf\_578\_278\_rev 5', mRNA sequence  
m gi|117541691|gb|EG873136.1|EG873136 EST\_ssal\_eve\_34015 ssaleve thyroid Salmo salar cDNA Salmo salar cDNA clone ssal\_eve\_546\_075\_fwd 3', mRNA sequence  
n gi|45304023|gb|CK874392.1|CK874392 SGP135598 Atlantic salmon Brain cDNA library Salmo salar cDNA clone HJ4-0408 5', mRNA sequence  
o gi|117847756|gb|EG920452.1|EG920452 EST\_ssal\_evf\_59305 ssalevf mixed\_tissue Salmo salar cDNA Salmo salar cDNA clone ssal\_evf\_579\_266\_fwd 3', mRNA sequence  
p gi|117545651|gb|EG877096.1|EG877096 EST\_ssal\_eve\_20747 ssaleve thyroid Salmo salar cDNA Salmo salar cDNA clone ssal\_eve\_528\_080\_fwd 3', mRNA sequence  
q gi|117425130|gb|EG757354.1|EG757354 EST\_ssal\_sjb\_6022 ssalsjb mixed\_tissue Salmo salar cDNA Salmo salar cDNA clone ssal\_sjb\_013\_311\_fwd 3', mRNA sequence  
r gi|84204123|gb|DW177669.1|DW177669 SGP304267 Atlantic salmon Brain II cDNA library Salmo salar cDNA clone SS0083F10 5', mRNA sequence  
s gi|84204253|gb|DW177735.1|DW177735 SGP304348 Atlantic salmon Brain II cDNA library Salmo salar cDNA clone SS0085B10 5', mRNA sequence  
t gi|84206797|gb|DW179191.1|DW179191 SGP306093 Atlantic salmon Brain II cDNA library Salmo salar cDNA clone SS0189B01 5', mRNA sequence  
u gi|84204390|gb|DW177805.1|DW177805 SGP304436 Atlantic salmon Brain II cDNA library Salmo salar cDNA clone SS0086H03 5', mRNA sequence  
v gi|84206123|gb|DW178789.1|DW178789 SGP305585 Atlantic salmon Brain II cDNA library Salmo salar cDNA clone SS0176A02 5', mRNA sequence  
w gi|117856543|gb|EG929239.1|EG929239 EST\_ssal\_evf\_31473 ssalevf mixed\_tissue Salmo salar cDNA Salmo salar cDNA clone ssal\_evf\_541\_304\_fwd 3', mRNA sequence  
x gi|117856558|gb|EG929254.1|EG929254 EST\_ssal\_evf\_31486 ssalevf mixed\_tissue Salmo salar cDNA Salmo salar cDNA clone ssal\_evf\_541\_310\_fwd 3', mRNA sequence  
y gi|117545649|gb|EG877094.1|EG877094 EST\_ssal\_eve\_20745 ssaleve thyroid Salmo salar cDNA Salmo salar cDNA clone ssal\_eve\_528\_079\_fwd 3', mRNA sequence  
z gi|117467752|gb|EG799971.1|EG799971 EST\_ssal\_evd\_56757 ssalevd thymus Salmo salar cDNA Salmo salar cDNA clone ssal\_evd\_576\_209\_fwd 3', mRNA sequence  
A gi|117531833|gb|EG863278.1|EG863278 EST\_ssal\_eve\_56241 ssaleve thyroid Salmo salar cDNA Salmo salar cDNA clone ssal\_eve\_576\_251\_fwd 3', mRNA sequence  
B gi|117497069|gb|EG829286.1|EG829286 EST\_ssal\_eve\_41650 ssaleve thyroid Salmo salar cDNA Salmo salar cDNA clone ssal\_eve\_556\_250\_fwd 3', mRNA sequence  
C gi|117470846|gb|EG803065.1|EG803065 EST\_ssal\_evd\_278 ssalevd thymus Salmo salar cDNA Salmo salar cDNA clone ssal\_evd\_002\_055\_fwd 3', mRNA sequence  
D gi|117846960|gb|EG919656.1|EG919656 EST\_ssal\_evf\_58587 ssalevf mixed\_tissue Salmo salar cDNA Salmo salar cDNA clone ssal\_evf\_578\_278\_fwd 3', mRNA sequence  
E gi|117523737|gb|EG855464.1|EG855464 EST\_ssal\_eve\_16110 ssaleve thyroid Salmo salar cDNA Salmo salar cDNA clone ssal\_eve\_521\_332\_fwd 3', mRNA sequence  
F gi|117565874|gb|EG886850.1|EG886850 EST\_ssal\_evf\_45066 ssalevf mixed\_tissue Salmo salar cDNA Salmo salar cDNA clone ssal\_evf\_560\_089\_fwd 3', mRNA sequence  
G gi|117868085|gb|EG940781.1|EG940781 EST\_ssal\_evf\_24272 ssalevf mixed\_tissue Salmo salar cDNA Salmo salar cDNA clone ssal\_evf\_532\_043\_fwd 3', mRNA sequence  
H gi|117544418|gb|EG875863.1|EG875863 EST\_ssal\_eve\_19636 ssaleve thyroid Salmo salar cDNA Salmo salar cDNA clone ssal\_eve\_526\_275\_fwd 3', mRNA sequence  
I gi|117445613|gb|EG777836.1|EG777836 EST\_ssal\_evd\_4435 ssalevd thymus Salmo salar cDNA Salmo salar cDNA clone ssal\_evd\_504\_221\_fwd 3', mRNA sequence  
J gi|84204499|gb|DW177860.1|DW177860 SGP304503 Atlantic salmon Brain II cDNA library Salmo salar cDNA clone SS0088C04 5', mRNA sequence

5 SNPs detected

A B C D E F G H I J K L M N O P Q R S T U V W X Y Z a b c d e f g h i j k l m n o p q r s t u v w x y z A B C D E F G H I J K L M N O P Q R S T U V W X Y Z a b c d e f g h i j k l m n o p q r s t u v w x y z A B C D E F G H I J  cosegregation weighted

199 . . A A A A A . A . A A A - A A A A A A A - - A A A A - - - - A A A . A A - - - A A A . . . A . . . . . . . . . . . . . . . . . . . . . . . . . . . . . . . . . . . . . . . . . . . . . . . . . . . . . . . . . . . . . . . . . . .   4/5 27.37
200 . . A A A A A . A . A A A - A A A A A A A - - A A A A - - - - A A A . A A - - - A A A . . . A . . . . . . . . . . . . . . . . . . . . . . . . . . . . . . . . . . . . . . . . . . . . . . . . . . . . . . . . . . . . . . . . . . .   4/5 27.37
201 . . A A A A A . A . A A A - A A A A A A A - - A A A A - - - - A A A . A A - - - A A A . . . A . . . . . . . . . . . . . . . . . . . . . . . . . . . . . . . . . . . . . . . . . . . . . . . . . . . . . . . . . . . . . . . . . . .   4/5 27.37
617 . . . . . . . . . . . . . . . G G C C C G G G G C C C G G G G C C G G G G G G G C C G C . G G C C G G . . . . . . . . . . . . . . . . . . . . . . . . . . . . . . . . . . . . . . . . . G . . . . . . . . . . . . . . . . . . . . .   1/5 6.32
1460 . . . . . . . . . . . . . . . . . . . . . . . . . . . . . . . . . . . . . . . . . . . . . . . . . . . . . . . . . . . - - - - - - - - - - - - - - - . - - - - - - - . - - - - - - A A - A A - - - - - - - - - - A A A A - - - A A -   4/5 37.19
